# Increased Vaccination Coverage among Adolescents and Young Adults in the District of Palermo as a Result of a Public Health Strategy to Counteract an ‘Epidemic Panic’

**DOI:** 10.3390/ijerph15051014

**Published:** 2018-05-17

**Authors:** Claudio Costantino, Vincenzo Restivo, Gianmarco Ventura, Claudio D’Angelo, Maria Angela Randazzo, Nicolò Casuccio, Mario Palermo, Alessandra Casuccio, Francesco Vitale

**Affiliations:** 1 Department of Science for Health Promotion and Mother to Child Care “G. D’Alessandro”, University of Palermo, 90127 Palermo, Italy; claudio.costantino01@unipa.it (C.C.); vincenzo.restivo@unipa.it (V.R.); alcamese@gmail.com (G.V.); francesco.vitale@unipa.it (F.V.); 2 Department of Medical Prevention, Local Health Unit of Palermo, 90100 Palermo, Italy; claudiodangelo847@alice.it (C.D.); randazzo.marilina@libero.it (M.A.R.); trudeau@libero.it (N.C.); 3 Sicilian Health Department, Public Health and Environmental Risks Service, 90127 Palermo, Italy; mario.palermo@regione.sicilia.it

**Keywords:** meningitis, vaccination campaign, mass media, outbreak

## Abstract

During the summer of 2016 four cases of invasive meningococcal disease in rapid succession among young adults in the district of Palermo, Italy, resulting in one death, were widely reported by local and national mass media. The resultant ‘epidemic panic’ among the general population overloaded the vaccination units of the Palermo district over the following months. Strategies implemented by the Sicilian and local public health authorities to counteract ‘meningitis fear’ included the following: (a) extension of active and free-of-charge anti-meningococcal tetravalent vaccination from age class 12–18 to 12–30 years old; (b) implementation of vaccination units during normal clinic hours in rooms tailored for vaccine administration; (c) development of informative institutional tools and timely communication throughout local mass media to reassure the general population. In 2016, an increase in the anti-meningococcal coverage was observed in the Palermo district (+18% for 16-year-olds and +14% for 18-year-olds) and at the regional level (+11.2% and +13.5%, respectively). Concurrent catch-up of other recommended vaccinations for age (diphtheria-tetanus-pertussis-poliomyelitis and papillomavirus) resulted in a further increase of administered doses. The fear of meningitis, managed by the Sicilian public health authorities, had positive impacts in terms of prevention. In particular, the communication strategies that were adopted contributed to educating Sicilian young adults about vaccination issues.

## 1. Introduction

In 2016, in Italy, *Neisseria meningitidis* was the most frequent cause of invasive meningococcal disease (IMD). The incidence rate was 0.38/100,000, which was a slight increase compared to previous years (0.23 in 2012, 0.29 in 2013, 0.27 in 2014 and 0.31 per 100,000 in 2015) [1]. In Europe, northern regions (Lithuania, UK, Ireland and Iceland) were the most affected, with incidence rates of more than 1 case per 100,000 [2]. However, the incidence rates appeared to be decreasing where vaccination campaigns were carried out since the introduction of the vaccination against serotype C in 1999 [3].

Vaccination is recognised as the best way to counteract the spread of the bacterium and, consequently, the development of epidemic outbreaks [4]. Since 2016, the Italian vaccination schedule has consisted of one administration of conjugate anti-meningococcal C vaccination at the age of 13–15 months and a second dose, with a tetravalent conjugate formulation (ACW135Y), from the age of 11 to 18 years old [5]. Anti-meningococcal tetravalent vaccination coverage reported by the Italian Health Department during 2016 was 19.69% among 16-year-olds and 7.64% among 18-year-olds [6,7].

As shown in Table 1, between June and August 2016, four cases of invasive meningococcal disease occurred in the city of Palermo, Italy, within a very short timeframe. The first affected subjects were two young girls who had attended nightlife venues in Palermo during the previous weeks for work-related reasons. The first case, involving *N. meningiditidis* serogroup C, was a 22-year-old female who died within 24 h from the onset of symptoms. The second case, a 23-year-old girl affected by *N. meningiditidis* serogroup B, received therapy that resulted in healing without serious typical meningitis complications such as hearing or vision loss or loss of limbs. A third case, which was resolved after hospitalisation, was caused by the *N. meningiditidis* W135 strain; the patient was an Eritrean migrant adolescent who disembarked at the Palermo harbour. Symptoms again were resolved after hospital intervention. Finally, in August 2016, there was a fourth case involving a 22-year-old female who was on holiday in Sicily and was treated and released without serious consequences from a Tuscan hospital.

Although only one death occurred, the rapid succession of the four cases among young adults, in addition to the first two affected girls attending nightclubs, gained extraordinary prominence in the local and national mass media. The impact on the imagination of the population, emphasised by mass media, resulted in an ‘epidemic panic’ that overloaded the vaccination units of the Palermo district over the following months.

The aim of this study was to assess whether such an emergency, together with communication strategies and measures provided by the Sicilian public health authorities, could lead not only to an increase in the anti-meningococcal vaccination coverage in the target population but also to a positive effect on vaccine compliance in the general population and increased coverage of other vaccinations of the Sicilian schedule.

## 2. Materials and Methods

The Sicilian region is the largest area of Italy and represents the fourth-most populated among the twenty Italian regions. The Sicilian Health Department has divided the 9th Sicilian District into nine corresponding local health units (Agrigento, Caltanissetta, Catania, Enna, Messina, Palermo, Ragusa, Siracusa and Trapani). Data on the vaccination coverage for all included vaccines in the Sicilian vaccination schedule are available yearly for the regional and district levels.

After each meningitis case occurred, the Prevention Department of the Palermo Local Health Unit promptly arranged the routine epidemiological investigation of each suspected case. Close contacts of the affected subjects were treated with ciprofloxacin for anti-meningococcal prophylaxis and occasional contacts were promptly informed about the precautions to be taken in case of symptoms. Laboratory tests were performed to confirm the diagnosis and to determine the aetiological agent. Once the necessary information had been obtained, punctual communication of the results by public health authorities was carried out through institutional channels and mass media in order to inform the general population and to reassure the public about the risk of contracting the disease [8,9].

Despite the massive information campaign of the local health authorities, the local and national media coverage of the cases was disproportionate, dedicating a large amount of space for news regarding the ‘outbreak’ on every platform, including newspapers, television, websites and social networks. In the following days, news and updates about each suspected case were disseminated without waiting for the laboratory confirmation of the case and without a response from the local health authorities to the potentially inaccurate information reported. This situation caused an unjustified fear of contagion among the general population and the incorrect notion that it was a real meningitis outbreak. As a consequence, subjects of all ages, but above all adolescents and young adults, swamped vaccination services, not only in the city of Palermo but also in the entire district to request anti-meningococcal vaccinations [10].

As shown in Table 2, until June 2016, according to the Sicilian vaccination schedule, the anti-meningococcal vaccination was actively offered and was free-of-charge at 13–15 months (anti-meningococcal C conjugate monovalent vaccine) and 12–18 years (anti-meningococcal ACW135Y conjugate tetravalent vaccine) of age. Subjects of age >18 years who requested the tetravalent vaccination could be vaccinated but there was a co-payment, i.e., half of the price of the vaccine was paid by the Sicilian Health Department and half by the patient [11].

One of the first actions implemented by Sicilian public health authorities was, in July 2016, the extension of the active and free vaccination offer of the tetravalent anti-meningococcal vaccine to subjects aged between 18 and 30 years [12]. Subsequently, a further decree issued by the Sicilian Health Department extended the vaccination offer to health professionals of emergency departments and to anyone who, for study or work reasons, had to go to the Tuscany region, where the notifications of IMD were constantly increasing from 2015 [13].

To counteract the growing number of requests for vaccination by the general population, the vaccination services of the Palermo district extended its working hours and days and healthcare workers were recalled from holidays. Moreover, in collaboration with the School of Hygiene and Preventive Medicine of the University of Palermo, Italy, twelve medical residents were recruited to the staff of the vaccination services to support the extra workload generated. Furthermore, during the second semester of 2016 (from July until December), all subjects attending vaccination services for the anti-meningococcal tetravalent vaccine were offered catch-up vaccinations of anti-diphtheria-tetanus-pertussis + poliomyelitis (dTpa + IPV-diftheria, tetanus, pertussis and inactivate poliomyelitis) and the anti-papillomavirus (HPV) vaccinations. Also, in order to counteract the ‘epidemic panic’, numerous interviews with local and national media by public health authorities took place throughout the summer, as well public announcements to explain the real risk. In addition, both on the official website of the local health unit and in the main sites where youth gathered (e.g., schools, public places and nightlife venues), informative material on preventive measures for meningitis infection was disseminated.

Data of the anti-meningococcal vaccination coverage for the two age groups (16 and 18 years old) for which the Regional Health Department produces an annual report, as requested by National Health Department, were reported. Coverage rates for the eligible cohort were calculated for 2015 and 2016 and differences in the anti-meningococcal vaccination coverage among the various Sicilian local health units were also analysed. In Sicily, the 16-year-old cohort for the year 2015 was made up of 53,162 subjects and in 2016, 51,478 adolescents. The 18-year-old cohort included 53,886 subjects in 2015 and 53,002 in 2016 [14]. The Chi-square test was used to evaluate the statistical significance of differences in the vaccination coverage among the local health units and at a regional level. Finally, the number of administered doses for the other two vaccines (anti-diphtheria-tetanus-pertussis-poliomyelitis and anti-papillomavirus) offered actively and free-of-charge in Sicily to adolescents was evaluated for 2015 and 2016.

This study was approved by the Ethics Committee of the Policlinico ‘Paolo Giaccone’ Hospital (Palermo 1).

## 3. Results

As reported in Table 3, from 2015 to 2016 in Sicily, the overall coverage rate for the anti-meningococcal vaccination in the 16-year-old cohort showed an increase from 39.8% to 54.4% (+14.6%). In eight of the nine Sicilian local health units, the coverage increased, with the highest growth achieved in the local health unit of Agrigento (+29.7%), Ragusa (+21.4%) and Palermo (+8%).The local health Unit of Palermo also reached the highest coverage rate for the 16-year-old cohort at the regional level (78.2%).

Positive results were also recorded in the 18-year-old cohort with an increasing vaccination coverage from 30.2% in 2015 to 45.1% in 2016 (+14.9%). The local health unit of Agrigento confirmed the highest increase (+33.6%), followed by Ragusa, Siracusa, Enna, Trapani and Palermo (+19.8%, +18.1%, +15.7%, +14.4% and +14%, respectively). The highest anti-meningococcal vaccination rate among the 18-year-old cohort was observed in the Palermo district (71.2%). All increases were statistically significant.

**Figure 1:**
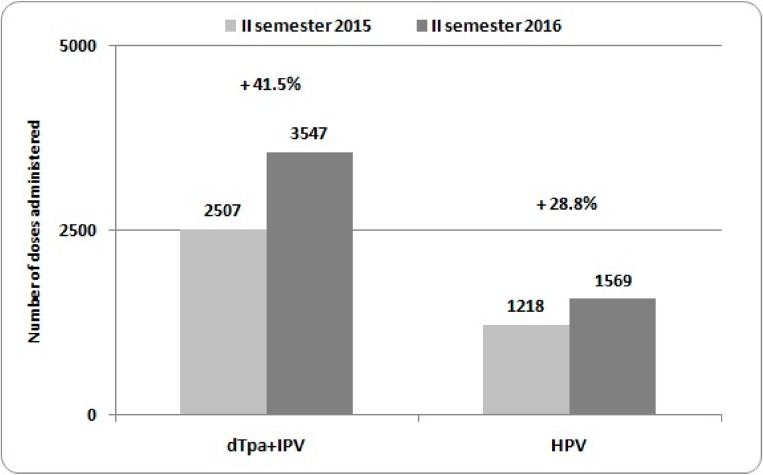
Number of doses of dTpa + IPV and HPV vaccines administered in the Local Health Unit of Palermo among 12–18 years old adolescents during the second semester of 2015 and 2016, with the percentage changes.

Figure 1 shows the number of single-dose anti-diphtheria-tetanus-pertussis-poliomyelitis (dTpa + IPV) and anti-papillomavirus (HPV) vaccines carried out in the district of Palermo during the second semester of 2015 and 2016(from the 1st of July until the 31th of December). An increase in the number of administered doses of dTpa + IPV (+29.3%) and HPV (+22.4%) vaccinations was observed.

## 4. Discussion

The Sicilian public health authorities responded promptly to a perceived outbreak of meningitis in the following ways: expanding the active and free offer of the anti-meningococcal vaccine C and ACW135Y from 12–18 to 12–30 years; providing information in public places; communication through institutional websites and social media; providing punctual counselling activity at the vaccination units to educate young adults about vaccines; and making appropriate vaccine recalls or administration according to the Sicilian vaccination schedule.

The data analysed in the present study showed a consistent increase in the anti-meningococcal vaccination rates in the two cohorts of 16- and 18-year-olds. As expected, in the local health unit of Palermo, where four meningitis cases occurred, there was a higher vaccination rate in both cohorts. In eight of nine Sicilian local health units, there was an increase in the vaccination coverage between 2015 and 2016. However, the highest incremental values were found in the local health unit of Agrigento, probably for two reasons: firstly, the second case of meningitis involved a girl residing in the Agrigento local health unit, secondly, a significant percentage of the population residing there, often go to Palermo for study or work reasons. Similar considerations could be applied to the local health units of Trapani and Caltanissetta, where many young adults often go to the Palermo.

The panic among the general population probably originated from the fear that what occurred in the Tuscany Region in 2015, where there has been a noticeable increase in cases of IMD due to a particularly aggressive Neisseria strain, could be repeated in Sicily [13]. Specifically, in Tuscany, IMD cases increased from 0.08 per 100,000 in 2012 to 1.98 per 100,000 in January–February 2016. This increase affected all age groups but particularly adolescents and young adults. However, in Sicily, unlike in other Italian regions, the incidence rate of IMD remained essentially unchanged in all age groups [1,15]. Moreover, the main aetiological agent of IMD in 2016 in Sicily was *S. Pneumoniae* [1,15].

The alarm of a potential epidemic was, therefore, unjustified, as correctly reiterated by the public health authorities. On the other hand, as reported by other authors, suitable communication can make the difference in the management of a possible epidemic outbreak [16,17].

The demand for vaccination services in the days immediately following the first two cases of meningitis confirmed that communication to the general population on relevant public health topics should not be left to journalists who are not experts in the field.

This event allowed counselling to be given to adolescents and young adults on the anti-meningococcal vaccination and also allowed for the catch-up of any vaccinations not carried out previously (i.e., anti-HPV, anti-dTpa + IPV). The observed increase in anti-meningococcal vaccination coverage and of the other vaccinations was a positive effect of the ‘epidemic of panic’.

In similar circumstances, the public health authorities should operate on at least two levels. One, the institutional level: adopting effective communication strategies to provide timely and effective information on appropriate prevention measures and regulated access for the population to receive vaccination services; and two, at the local level, where healthcare workers of the vaccination services can target counselling to adolescents and young adults in order to promote a greater confidence in vaccination.

## 5. Conclusions

After the occurrence of the four cases of meningococcal disease during summer 2016 in Palermo, we observed a significant increase in the anti-meningococcal vaccination coverage in each local health unit and at a regional level. Concurrent catch-up of other recommended vaccinations for age (diphtheria-tetanus-pertussis-poliomyelitis and papillomavirus) resulted in a further increase of administered doses. 

Communication strategies adopted by the Sicilian public health authorities in the management of the “epidemic of panic”, had a positive impacts educating young adults about vaccination issues.

In the future, the media should address health issues by using appropriate medical expert to inform the population of the risks and dangers through risk-minimisation strategies. Moreover, public health authorities should be aware of new communication platforms of which adolescents and young adults are the main users.

## Figures and Tables

**Table 1:** Brief summary of invasive meningococcal disease cases observed in the Palermo District during 2016 summer.

ID	Notification Date	Age (Years Old)	Serotype	Outcome	Tight Contact Identified during Previous 4–6 Weeks
01	13 June 2016	23	C	Death	Nightlife co-workers, Japanese course colleagues, friends/family
02	7 July 2016	22	B	Hospitalization, healing	Nightlife co-workers, family, aunt, boyfriend
03	20 July 2016	15	W135	Hospitalization, healing	Travel companions, health care workers
04	5 August 2016	22	Unknown	Hospitalization, healing	Family, travel companions

**Table 2:** Sicilian Vaccination Schedule at 30 June of 2016 [11]. (In Sicily, according to the amount of Difteria and Pertussis antigen in the vaccine there are two different formulation with a DTPa for children under two years of life and a dTpa for all subjects older than two years old).

Vaccine	3rd Month (Since 61st Day of Life)		After 1 Month Since Hexavalent/PCV13 and Rota	5th Month (Since 121st day of Life)		After 1 Month Since Hexavalent/PCV13 and Rota	Since 1 Month Since the Second Meningo B Dose	11th–12th Month		13th–15th Month	Since 1 Month from MMRV	5th–6th Year of Life	12th Year of Life	15th–18th Year of Life	19th–64th Year of Life	>65 Years
Diphtheria, Tetanus and Pertussis	DTPa	HEXAVALENT		DTPa	HEXAVALENT			DTPa	HEXAVALENT			dTpa + IPVordTpa/IPV		dTpa + IPVordTpa/IPV	dTpaevery 10 years	
Polio	IPV			IPV				IPV								
Hepatitis B	HBV			HBV				HBV								
Haemophilusinfluenzae type B (Hib)	HiB			HiB				HiB								
Pneumococcal (Conjugate)	PCV13			PCV13				PCV13		For high-risk subjects PCV13 (conjugate) e PPV 23 (polysaccharide)						PCV13/PPV23
Rotavirus	Rotavirus(oral)			Rotavirus (oral)												
Meningococcal B			Meningo B			Meningo B	Meningo B				Meningo B					
Meningococcal C										Meningo C						
Meningococcal ACW135Y													MeningoACW135Y			
Measles, Mumps, Rubella and Varicella										MMRV or MMR + V		MMRV or MMR + V				
Papillomavirus													HPV (Males and females)		(F) until 45 years	
															(M) until 26 years	
Seasonal Influenza							For high-risk subjects seasonal influenza vaccination									Seasonal influenza
Herpes Zoster															>50 anni se a rischio (altrepatologie)	Zoster

**Table 3:** Coverage with anti-meningococcal vaccine among 16 and 18 years old cohorts in the nine Local Health Unit of the Sicilian Districts (2015 vs. 2016).

Local Health Unit	16 Years Old Cohort			18 Years Old Cohort		
	Coverage 2015 (%)	Coverage 2016 (%)	Percentagepoint Change	Coverage 2015 (%)	Coverage 2016 (%)	Percentagepoint Change
Agrigento	42.7	72.4	29.7 *	29	62.6	33.6 *
Caltanissetta	53.6	49.9	−3.7	36.8	46.8	10 **
Catania	15.8	31.8	16 *	4.4	23.6	18.8 *
Enna	41.6	48.1	6.5 ***	33.3	49	15.7 *
Messina	30.1	35.1	5 **	24	22.2	−1.8
Palermo	60.2	78.2	18 *	57.2	71.2	14 *
Ragusa	59.4	80.8	21.4 *	43.9	63.7	19.8 *
Siracusa	35	48.1	13.1 *	20.1	38.2	18.1 *
Trapani	32.6	46.6	14 *	20.3	34.7	14.4 *
Overall	39.8	54.4	14.6 *	30.2	45.1	14.9 *

* *p*-value < 0.001; **p-value < 0.01; ***p-value < 0.05.
